# Comparison of Overall Survival Between Invasive Lobular Breast Carcinoma and Invasive Ductal Breast Carcinoma: A Propensity Score Matching Study Based on SEER Database

**DOI:** 10.3389/fonc.2020.590643

**Published:** 2020-12-22

**Authors:** Ciqiu Yang, Chuqian Lei, Yi Zhang, Junsheng Zhang, Fei Ji, Weijun Pan, Liulu Zhang, Hongfei Gao, Mei Yang, Jieqing Li, Kun Wang

**Affiliations:** ^1^ Department of Breast Cancer, Cancer Center, Guangdong Provincial People’s Hospital, Guangdong Academy of Medical Sciences, Guangzhou, China; ^2^ Department of Breast and Thyroid Surgery, The Eighth Affiliated Hospital, Sun Yat-sen University, Shenzhen, China

**Keywords:** invasive lobular carcinoma, invasive ductal carcinoma, SEER program, overall survival, breast cancer

## Abstract

**Objective:**

Invasive lobular carcinoma (ILC) and invasive ductal carcinoma (IDC) account for most breast cancers. However, the overall survival (OS) differences between ILC and IDC remain controversial. This study aimed to compare nonmetastatic ILC to IDC in terms of survival and prognostic factors for ILC.

**Methods:**

This retrospective cohort study used data from the Surveillance, Epidemiology and End Results (SEER) Cancer Database (www.seer.cancer.gov). Women diagnosed with nonmetastatic ILC and IDC between 2006 and 2016 were included. A propensity score matching (PSM) method was used in our analysis to reduce baseline differences in clinicopathological characteristics and survival outcomes. Kaplan-Meier curves and log-rank test were used for survival analysis.

**Results:**

Compared to IDC patients, ILC patients were diagnosed later in life with poorly differentiated and larger lesions, as well as increased expression of estrogen receptors (ERs) and/or progesterone receptors (PRs). A lower rate of radiation therapy and chemotherapy was observed in ILC. After PSM, ILC, and IDC patients exhibited similar OS (HR=1.017, p=0.409, 95% CI: 0.967–1.069). In subgroup analysis of HR-negative, AJCC stage III, N2/N3 stage patients, or those who received radiotherapy, ILC patients exhibited worse OS compared to IDC patients. Furthermore, multivariate analysis revealed a 47% survival benefit for IDC compared to ILC in HR-negative patients who received chemotherapy (HR=1.47, p=0.01, 95% CI: 1.09–1.97).

**Conclusions:**

Our results demonstrated that ILC and IDC patients had similar OS after PSM. However, ILC patients with high risk indicators had worse OS compared to IDC patients by subgroup analysis.

## Introduction

Invasive lobular carcinoma (ILC) is the second most common histological subtype of invasive breast cancer and contributes to 5%–15% of all breast cancer cases (1–4). The incidence of ILC is increasing, especially in women older than fifty (5, 6). At present, most of the systemic therapy decisions for ILC are derived from randomized clinical trials based on invasive ductal carcinoma (IDC), which may explain why the St Gallen International Expert Consensus guidelines and the National Comprehensive Cancer Network (NCCN) still recommend that ILC be treated with the same treatment paradigms as IDC; however, ILC has many unique features.

Compared to IDC, ILC has better prognosis, being almost invariably positive for hormone receptors, with low histological grade, negative for HER2 and with a generally good response to endocrine therapy (1, 3). In general, ILC tends to grow in a multicentric or multifocal pattern, which makes resection for negative margins difficult when performing breast-conserving surgery (7–10). In addition, ILC patients tend to be at risk for distant recurrence for greater than 5–10 years (11) and have an unusual metastatic site with involvement of the gastrointestinal tract, pelvic organs and peritoneal sites (12, 13). Therefore, the clinical response of ILC has unique aspects and deserves special attention. ILC is considered to be less chemo-sensitive for either adjuvant or neoadjuvant chemotherapy compared to IDC, which is thought to be mediated by molecular features, such as low-grade and estrogen receptor (ER) positivity (1, 3, 4, 9, 14, 15).

There are conflicting data regarding the prognosis between ILC and IDC. Long-term outcomes for ILC have been reported as worse (16–19), no different (20–23), and better (3, 4, 24, 25) than for IDC. Whether differences exist with respect to disease-free survival (DFS) and overall survival (OS) between ILC and IDC is still controversial. Furthermore, differences in prognosis between ILC and IDC with the same molecular subtypes are not clear. Our study used a large database to further compare survival between IDC and ILC across a range of subgroups and to identify prognostic factors for early breast cancer patients with ILC.

## Materials and Methods

### SEER Database

This study cohort employed data from the National Cancer Institute’s Surveillance, Epidemiology, and End Results (SEER) 18 tumor registry database that was updated in November 2016. The SEER program registries contain population demographics, tumor characteristics, nodal staging, surgery information, vital status, and follow-up information from 18 geographic regions with more than 3 million patients, covering approximately 28% of the U.S. population. The database emphasizes quality control and stipulates a less than five percent error rate (26). We defined IDC patients according to the International Classification of Diseases (ICD) histological code 8500/3 and ILC patients according to the ICD histological code 8520/3.

We were granted permission to access the cancer files from the SEER program along with other treatment information, including chemotherapy and radiation therapy with a reference number of 19952-Nov2018. The requirement for informed consent was waived because personal information of patients was not involved.

### Case Selection

We first identified 288,216 IDC patients and 30,190 IDC patients from 2006 to 2016 according to the following criteria: female breast cancer patients aged 18–90 years old; primary cancer only; unilateral and known laterality; ductal and/or lobular carcinoma; availability of detailed information about grade, T/N stages, ER status, and PR status; and availability of detailed data about survival (**Table 1**). We used PSM to equate the two groups, and the final cohort consisted of 58,398 patients, 29,199 IDC patients, and 29,199 ILC patients.

**Table 1:** Stepwise inclusion and exclusion counts.

Removal criterion	Removed	Remaining
IDC or ILC patients	0 (0.0%)	1,097,908
Exclude patients with multiple primary lesions	327,616 (28.84%)	770,292
Exclude men	5,122 (0.01%)	765,170
Exclude patients younger than <18 years and >90 years	7,798 (0.01%)	757,372
Exclude patients with bilateral involvement or unknown laterality	1,448 (0.001%)	755,924
Exclude patients who did not receive a mastectomy or lumpectomy(exclude surgery unknown)	212506 (28.11%)	543,418
Exclude patients who did not have a histologically confirmed diagnosis	25,245 (0.05%)	518,203
Exclude patients with borderline or unknown ER	27,205 (0.05%)	490998
Exclude patients with borderline or unknown PR	5,700 (0.01%)	485,298
Exclude patients whose disease is not stage I–III	17,296 (0.04%)	468,002
Exclude those patients who is T0/Tis/Tx/T umknown and Nx/N umknown	886 (0.002%)	467,116
Exclude patients diagnosed in nursing home/hospice or by autopsy/death record only <3 month	11,265 (0.02%)	455851
2006–2016 patients	137,445 (30.15%)	318,406
Final data set	IDC 288216(90.52%) ILC 30190(9.48%)	

To eliminate differences in treatment measure and to ensure corresponding follow-up, this research period was from 2006 to 2016, and the cut-off date was December 31, 2016. Tumor and nodal stage were coded in line with the American Joint Committee on Cancer (AJCC) staging system for breast, using the 6th edition criteria for patients diagnosed from 2006 to 2009 and the 7th edition criteria for patients diagnosed from 2010 to 2016. Undifferentiated, anaplastic and poorly differentiated grade cases were considered grade III cases.

### Data and Statistical Analysis

Differences in the characteristic variables between ILC and IDC were compared by Chi-square test. The multivariate relationship of tumor characteristic variables and survival outcomes were examined by Cox proportional hazard model. Statistical significance was set at 0.05. OS was used as the survival endpoint in this survey and was analyzed using the Kaplan–Meier method. OS was defined as the time between confirming breast cancer to any cause of death. The log-rank test was utilized to calculate the hazard ratio (HR) and 95% confidence interval (95% CI) for OS. Our study utilized the propensity score matching method to diminish baseline differences in clinicopathological characteristics and survival outcome. Cases were 1:1 matched between ILC and IDC patients in accordance with age, histological grade, tumor stage, nodal stage, ER status, PR status, and so on. Statistical analysis was performed using Statistical Product and Service Solutions (SPSS) software (version 22; SPSS Inc., Chicago, USA). The propensity score matching method was calculated using the “MatchIt” package in R software (version 3.6.2, Synergy Software, Inc., Essex Junction, VT, USA).

## Results

### Patient Characteristics Between ILC and IDC

The SEER tumor registry database was used to identify 1,097,908 patients diagnosed with ILC and IDC. After selecting patients based on specific inclusion and exclusion criteria, the remaining 318,406 patients were included in our research. **Table 1** shows the patient selection process. Finally, 30,190 patients (9.48%) were assigned to the ILC group and 288,216 patients (90.52%) were assigned to the IDC group. **Table 2** summarizes the clinical characteristics of the ILC and IDC groups.

**Table 2:** Comparison of clinical characteristics between invasive lobular carcinoma (ILC) and invasive ductal carcinoma (IDC) group in unmatched population.

	IDC		ILC		P value
	N	%	N	%	
Age					<0.0001
<50	74,016	25.7	4,991	16.5	
≥50	214,200	74.3	25,199	83.5	
Race					<0.0001
White	226,764	78.7	25,580	84.7	
Black	31,450	10.9	2,550	8.4	
Asian or India	28,352	9.8	1,885	6.2	
Unknown	1,650	0.6	175	0.6	
Marital status					<0.0001
Married	166,265	57.7	12,979	42.7	
Unmarried	121,951	42.3	17,393	57.3	
Grade					<0.0001
Grade I	59,545	20.7	8,837	29.3	
Grade II	119,281	41.1	18,660	61.8	
Grade III	109,390	38.0	2,693	8.9	
AJCC stage					<0.0001
I	153,104	53.1	13,055	43.2	
II	102,685	35.6	11,859	39.3	
III	32,427	11.3	5,276	17.5	
T Stage					<0.0001
T1	182,552	63.3	15,241	50.5	
T2	86,705	30.1	10,491	34.7	
T3	12,673	4.4	4,013	13.3	
T4	6,286	2.2	445	1.5	
N Stage					<0.0001
N0	197,355	68.5	20,009	66.3	
N1	67,186	23.3	6,600	21.9	
N2	15,912	5.5	2,048	6.8	
N3	7,763	2.7	1,533	5.1	
ER					<0.0001
Negative	58,105	20.2	630	2.1	
Positive	230,111	79.8	29,560	97.9	
PR					<0.0001
Negative	86,960	30.2	4,809	15.9	
Positive	201,256	69.8	25,381	84.1	
Radiation therapy					<0.0001
None or unknown	125,374	43.3	13,482	44.7	
Yes	163,842	56.7	16,708	55.3	
Chemotherapy					<0.0001
None or unknown	156,985	54.5	19,915	66.0	
Yes	131,231	45.5	10,275	34.0	
Breast surgery					<0.0001
BCS	175,170	60.8	15,107	50.0	
Mastectomy	113,046	39.2	15,083	50.0	

People diagnosed with ILC tended to be older (median age of 63 years old in the ILC group versus 59 years old in the IDC group; p<0.0001), exhibit poorly differentiated and larger lesions, be ER/PR positive and were administered less radiation therapy and chemotherapy.

Given the surgical procedures, ILC had a higher percent of mastectomy compared to IDC cases (50.0% versus 39.2%, respectively). A lower rate of radiation therapy and chemotherapy was observed in the ILC group (55.3% versus 56.7%; 34.0% versus 45.5%, respectively; p<0.0001).

### Survival Outcomes Between ILC and IDC Group

Due to significant differences in clinical characteristics between ILC and IDC groups, our research used the propensity score matching method, based on age, histological grade, tumor stage, nodal stage, ER status, PR status, surgery type, chemotherapy, and radiation therapy, to reduce discrepancies in survival outcomes between the two groups. Each ILC patient was matched to one IDC patient. As shown in **Table 3**, both ILC and IDC groups comprised 29,199 patients with similar baseline clinicopathological characteristics for further analysis.

**Table 3:** Comparison of clinical characteristics between invasive lobular carcinoma (ILC) and invasive ductal carcinoma (IDC) groups after matching.

	IDC		ILC		P value
	N	%	N	%	
**Age**					
<50	4,914	16.8	4,914	16.8	
≥50	24,285	83.2	24,285	83.2	
**Grade**					1.000
Grade I	8,215	28.1	8,215	28.1	
Grade II	18,291	62.6	18,291	62.6	
Grade III	2,693	9.2	2,693	9.2	
**AJCC stage**					0.906
I	13,103	44.9	13,054	44.7	
II	11,407	39.1	11,456	39.2	
III	4,689	16.1	4,689	16.1	
**T Stage**					1.000
T1	15,221	52.1	15,221	52.1	
T2	10,368	35.5	10,368	35.5	
T3	3,177	10.9	3,177	10.9	
T4	433	1.5	433	1.5	
**N Stage**					1.000
N0	19,607	67.1	19,607	67.1	
N1	6,368	21.8	6,368	21.8	
N2	1,932	6.6	1,932	6.6	
N3	1,292	4.4	1,292	4.4	
**ER**					1.000
Negative	612	2.1	612	2.1	
Positive	28,587	97.9	28,587	97.9	
**PR**					1.000
Negative	4,463	15.3	4,463	15.3	
Positive	24,736	84.7	24,736	84.7	
**Radiation therapy**					1.000
None or unknown	13,205	45.2	13,205	45.2	
Yes	15,994	54.8	15,994	54.8	
**Chemotherapy**					1.000
None or unknown	19,404	66.5	19,404	66.5	
Yes	9,795	33.5	9,795	33.5	
**Breast surgery**					1.000
BCS	15,038	51.5	15,038	51.5	
Mastectomy	14,161	48.5	14,161	48.5	

**Figure 1:**
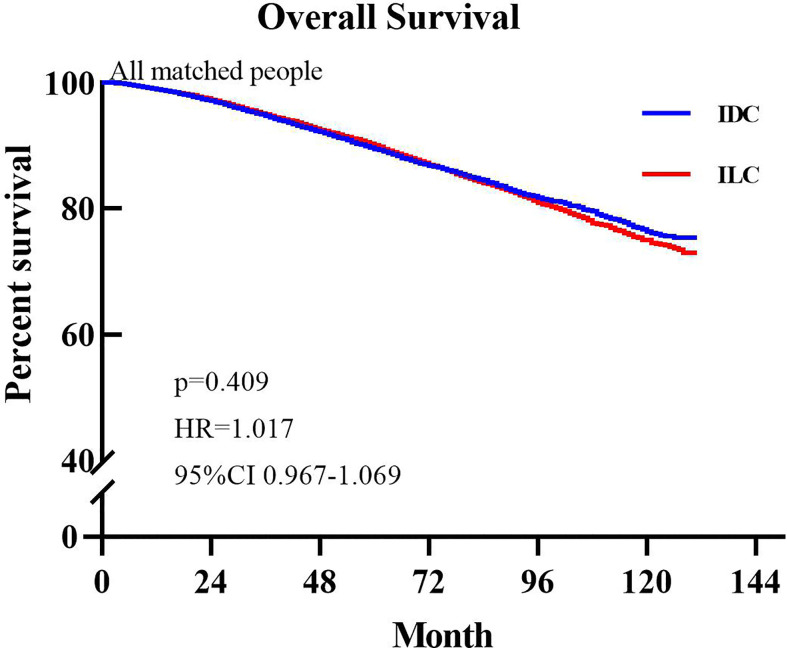
Kaplan-Meier survival curves of all matched patients.

The median OS was not reached in either group. During follow up, 2,999 patients (10.28%) in the ILC group died and 3,057 patients (10.46%) in the IDC group died. Based on comparison of the unmatched population database, IDC patients exhibited better OS (HR=1.045, P=0.025, 95% CI: 1.007–1.085) compared to ILC patients. **Figure 1** presents the Kaplan-Meier plots of overall survival in patients with ILC compared to those with IDC in the matched population. The median follow-up time for overall survival was 54 months (95% CI, 53.29–54.71 months) in the ILC cohort and 57 months (95% CI, 56.28–57.72 months) in the IDC cohort. Shown in **Figure 1**, ILC and IDC patients have similar OS (HR=1.017, P=0.409, 95% CI: 0.967–1.069).

### Survival Outcomes Between IDC and ILC Groups in the Subgroup Analysis

**Figure 2:**
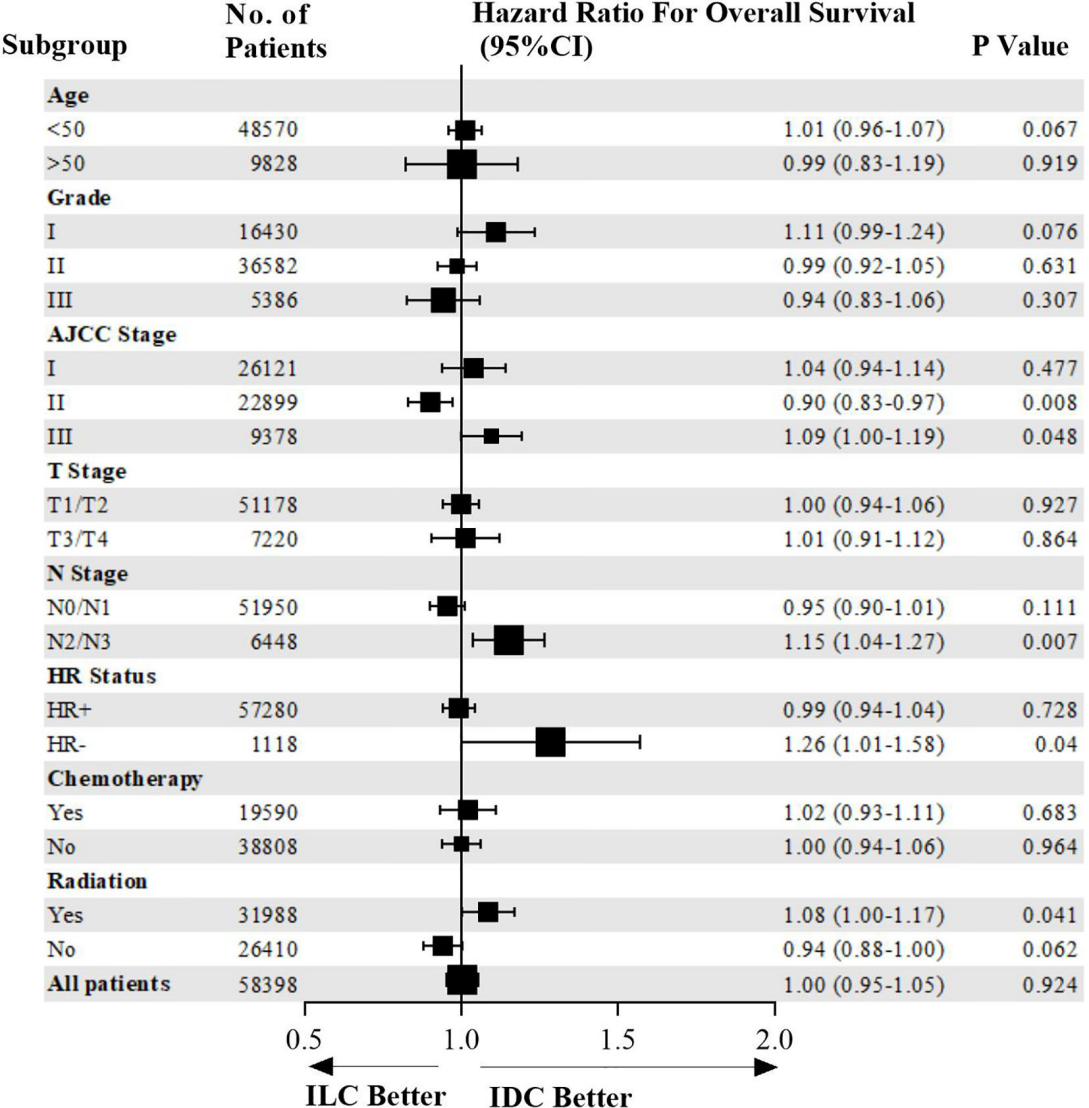
Forest plot of overall survival for patients in the entire cohort and subgroups.

**Figure 3:**
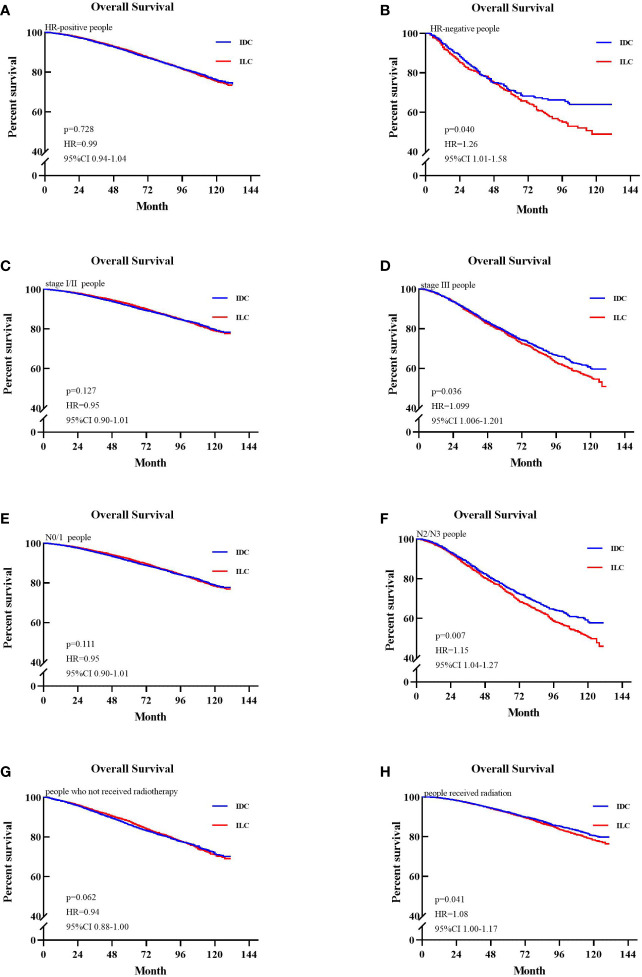
Kaplan-Meier survival curves of patients in different subgroups. **(A)** Overall survival (OS) between invasive lobular carcinoma (ILC) and invasive ductal carcinoma (IDC) group in the hazard ratio (HR)-positive cohort. **(B)** OS between ILC and IDC group in the HR-negative cohort. **(C)** OS between ILC and IDC group in the stage I/II cohort. **(D)** OS between ILC and IDC groups in the stage III cohort. **(E)** OS between ILC and IDC group in the node stage 0/1 cohort. **(F)** OS between ILC and IDC groups in the node stage 2/3 cohort. **(G)** OS between ILC and IDC groups of patients who not received radiotherapy. **(H)** OS between ILC and IDC groups of patients who received radiotherapy.

Overall survival of patients in the entire cohort and subgroups is shown in a Forest plot (**Figure 2**). ILC patients were associated with higher risk of mortality compared to IDC patients who were HR-negative, AJCC stage III, N2/N3 stage, or in those who received radiotherapy. Matched patients were divided into different subtypes to further examine factors affecting prognosis. In patients with positive hormone receptor, ILC and IDC groups exhibited similar OS (p=0.728, HR=0.99, 95% CI 0.94–1.04, **Figure 3A**). However, in patients with negative hormone receptor, the ILC group exhibited reduced OS compared to IDC patients (p=0.040, HR=1.26, 95% CI 1.01–1.58, **Figure 3B**). ILC presented a similar OS compared to IDC in AJCC stage I and II (p=0.127, HR=0.95, 95% CI 0.90–1.01, **Figure 3C**) but had worse OS in AJCC stage III (p=0.048, HR=1.09, 95% CI 1.00–1.19, **Figure 3D**). ILC had similar OS compared to IDC in N0 and N1 stage (p=0.111, HR=0.95, 95% CI 0.90–1.01, **Figure 3E**) but had worse OS in N2 and N3 stage (p=0.007, HR=1.15, 95% CI 1.04–1.27, **Figure 3F**). ILC had similar OS compared to IDC in patients who did not receive radiotherapy (p=0.062, HR=0.94, 95% CI 0.88–1.00, **Figure 3G**) but had worse OS in patients who received radiotherapy (p=0.041, HR=1.08, 95% CI 1.00–1.17, **Figure 3H**).

**Figure 4:**
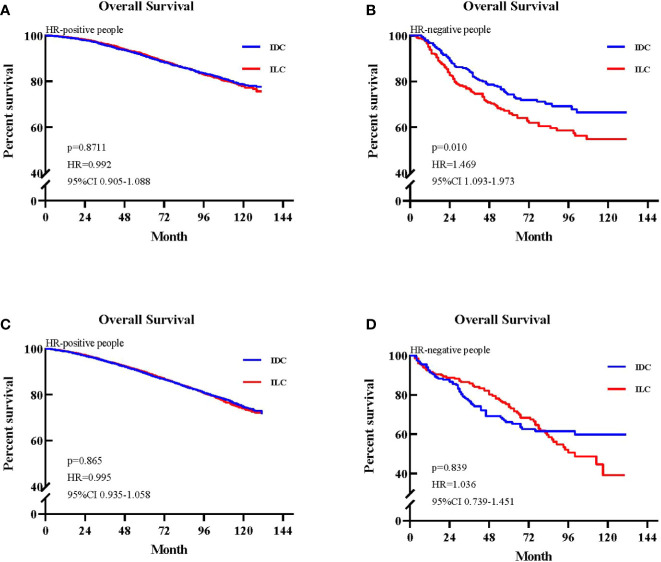
Kaplan-Meier survival curves of subgroups stratified by whether patients received chemotherapy. **(A)** Overall survival (OS) between invasive lobular carcinoma (ILC) and invasive ductal carcinoma (IDC) groups in the hazard ratio (HR)-positive cohort of patients who received chemotherapy. **(B)** OS between ILC and IDC groups in the HR-negative cohort of patients who received chemotherapy. **(C)** OS between ILC and IDC groups in the HR-positive cohort of patients who did not receive chemotherapy. **(D)** OS between ILC and IDC groups in the HR-negative cohort of patients who did not receive chemotherapy.

We stratified patients by treatment to further validate the differential prognosis by whether the patients received chemotherapy or not between the ILC and IDC cases. Among patients who received chemotherapy, the ILC group exhibited poorer OS compared with the IDC group in HR-negative patients (p=0.010, HR=1.47, 95% CI 1.09–1.97, **Figure 4B**). In contrast, the ILC and IDC groups presented similar OS in HR-positive patients (p=0.871, HR=0.99, 95% CI 0.91–1.09, **Figure 4A**). Among patients who did not receive chemotherapy, the ILC group had a similar OS compared to IDC group in both HR-positive and HR-negative patients (p=0.865, HR=1.00, 95% CI 0.94–1.06; p=0.839, HR=1.04, 95% CI 0.74–1.45, respectively. **Figures 4C, D**).

HER2 status is a very important prognostic and predictive factor in breast cancer. Therefore, we extracted patients with available HER2 status from the matched population, obtaining 39,684 patients in the HER2 cohort, of which 19,442 were IDC patients and 20,237 were ILC patients. Among these, there were 2,241 HER2+ patients (11.52%) and 17,206 HER2- patients (88.48%) in the IDC group and 981 HER2+ patients (4.85%) and 192,566 HER2- patients (95.15%) in the ILC group.

**Figure 5:**
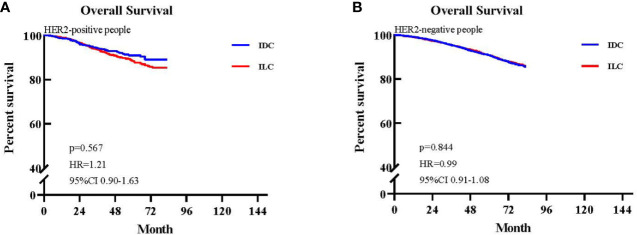
Kaplan-Meier survival curves of patients with available HER2 status. **(A)** Overall survival (OS) between invasive lobular carcinoma (ILC) and invasive ductal carcinoma (IDC) groups in the HER2-positive cohort. **(B)** OS between ILC and IDC groups in the HER2-negative cohort.

We further generated Kaplan–Meier survival curves and conducted a pairwise comparison between the two different HER2 statuses in IDC and ILC groups. We found that HER2 status was not a prognostic indicator for OS between IDC and ILC groups (HER2+: 92.99% vs. 94.60%, p>0.05; HER2-: 94.36% vs. 94.41%, p>0.05; **Figures 5A, B**).

## Discussion

ILC is the most common subtype of breast cancer that tends to have poor prognosis due to higher rates of lymph node metastasis compared to patients with IDC and tends to present with larger tumor size (27–29). However, ILC is also associated with favorable prognosis for patients with lower histologic grade, hormone receptor positivity and HER2 negative status (1, 3). Thus, the prognosis of ILC compared to IDC is still controversial. Our study was based on a retrospective analysis of a relatively large cohort of data retrieved from the population-based SEER database. After adjustment for patient and treatment characteristics, we found that the survival of ILC and IDC was equivalent in this analysis of outcomes for with two large patient cohorts. However, compared to IDC, we identified negative hormone receptor and positive lymph node statuses as adverse predictors for ILC.

In this study, we observed several differences in demographic and tumor characteristics between ILC and IDC in accordance with prior studies. ILC patients were older and had larger tumors than IDC patients. Moreover, ILC tumors were more often hormone receptor positive and of lower histological grade than IDC. The characteristics of these patients tended to be similar to prior studies reported for ILC (4, 11, 15, 30–32). The lower rate of chemotherapy can be attributed to the positivity of hormone receptors and low histological grade for most ILC patients, while the lower rate of BCS can be attributed to relatively larger tumor size. Furthermore, we found that the rate of lymph node positivity was higher in ILC in agreement with previous literature (15, 29, 32).

HER2 status is a very important prognostic and predictive factor in breast cancer. However, the majority of ILCs lack HER2 overexpression or amplification, and previous studies have reported that HER2 positivity occurs in 3%–5% of ILCs (30, 33). In our study, the rate of HER2 positivity was 4.85%, which is in accordance with data published previously. Furthermore, we found that HER2 status was not a prognostic indicator for OS HER2+ patients (HER2+: 92.99% vs. 94.60%, p>0.05; HER2-: 94.36% vs. 94.41%, p>0.05).

Previous studies comparing survival between IDC and ILC have reported conflicting results. Identifying 1034 patients who participated in six clinical trials from the M.D. Anderson Cancer Center, Cristofanilli et al. (4) demonstrated that ILC is characterized by lower rates of pathological response to neoadjuvant chemotherapy but better long-term outcomes compared to IDC. However, in a retrospective review of 171 ILC patients and 1,011 IDC patients, Fortunato et al. (23)found that ILC had no significant differences in outcomes compared to IDC and suggested that ILC can be treated similarly to IDC with good results. Wasif et al. used the SEER database to compare survival between ILC and IDC and reported that prognosis is better for patients with ILC than for those with IDC after stage matching (29). They reported that this outcome may be due to high expression of hormone receptors. However, they did not analyze the molecular subtype among ILC and IDC samples, and their study period was from 1993 to 2003. Thus, Adachi et al. (17) retrospectively analyzed the effect of chemotherapy in the Aichi Cancer Center among 1661 patients with luminal IDC and 105 patients with luminal ILC and found that luminal ILC had worse outcomes than luminal IDC. However, this study focused on the hormone receptor positive subtype. In our study, due to significant differences in clinical characteristics between ILC and IDC groups, we used the propensity score matching method to reduce discrepancies in survival outcomes. After each ILC patient was matched to one IDC patient, there was no difference in OS between the two groups. However, the prognosis of ILC was significantly worse than for IDC when stratified by negative hormone receptor and positive lymph node status. We believe that this may be related to ILC’s resistance to either adjuvant or neoadjuvant chemotherapy compared to IDC. In high risk patients, such as those who are hormone receptor negative and lymph node positive, adjuvant, or neoadjuvant chemotherapy was generally performed (ILC: 63% of HR-, 68% of LN+, 34% of all the ILC patients). Among patients who received chemotherapy, the ILC group had worse prognosis compared to the IDC group in HR-negative patients (HR 1.469; 95% CI 1.093–1.973; p=0.01), whereas ILC and IDC groups presented similar OS in HR-positive patients (HR 0.992; 95% CI 0.905–1.088; p=0.871).

The effect of neoadjuvant chemotherapy on ILC has been reported by several retrospective studies, with pCR rates ranging from 0-11% for ILC compared to 9%–25% for IDC (1, 4, 14, 15, 34). A total of 1,051 patients with ILC were included in a pooled analysis by Loibl et al. (22, 35, 36), and the end point was pathological complete response (pCR). There was a 6.2% pCR for ILC and 17.8% in HR negative and high-grade subgroups. A previous study also provided evidence that biologically aggressive ILCs (HR- and G3) achieved a higher pCR rate. However, the pCR rate is still lower in HR-negative ILC compared to HR-negative IDC (1). pCR, a surrogate endpoint marker, has been correlated with improved long-term outcomes, such as disease-free or overall survival (37–39), which could explain our result that HR-negative ILC exhibited worse OS than IDC. Furthermore, Huober et al. conducted a retrospective analysis to identify factors predicting relapse based on the GBG meta-database that includes five neoadjuvant trials (19). Their study demonstrated that the prognosis of patients with ILC was worse than IDC, even if all reached pCR. The reason for this result is that the majority of patients included in this study had triple negative breast cancer or HER2 positive breast cancer, in agreement with our study that ILC patients with HR-negative status have worse prognosis.

As mentioned above, ILC patients do not respond as well to neoadjuvant chemotherapy compared to IDC patients. This may explain a general assumption that ILC patients also have a poor response to adjuvant chemotherapy compared to IDC, but there is currently no data to support this notion. However, several studies have attempted to evaluate the effect of chemotherapy in ILC and reported no benefits followed by chemotherapy. Truin et al. (40) retrospectively examined the effect of chemotherapy in a multicenter cohort of postmenopausal patients with pure or mixed type ILC and found additional beneficial effects (10-year OS 66% vs 68%, p=0.45). Marmor et al. (41) also reported a similar result in patients with ER-positive, HER2-negative, stage I/II ILC who received endocrine therapy, demonstrating that they did not benefit from the addition of adjuvant chemotherapy. However, by analyzing 2,318 patients with ILC, Nonneville et al. (42) reported that ILC patients exhibited significant differences in DFS or OS benefit from chemotherapy in high-risk patients, such as those who were lymph node positive or presented with lympho-vascular invasion. In agreement with previous results, we identified negative HR, N2/N3, stage III, and receiving radiotherapy as predictors for long-term adverse outcomes in ILC. According to guidelines of the St Gallen International Expert Consensus and the National Comprehensive Cancer Network (NCCN), ILC patients are recommended the same systemic treatment as IDC patients. Systemic therapies include chemotherapy, radiotherapy, and endocrine therapy. Although previous studies have suggested that the sensitivity to radiotherapy is similar for ILC and IDC patients (43), the prognosis for ILC patients is still worse than IDC patients. Our explanation is that patients who receive radiotherapy tend to present positive lymph nodes and a larger tumor size, which are risk factors of worse survival. Chemotherapy is recommended for these patients with high risk. However, ILC patients are less sensitive to chemotherapy compared to IDC patients, which means that the treatment strategy of ILC may not completely be referred to that of IDC. ILC patients have their unique characteristics and might require additional stronger systemic treatments to improve survival. Previous studies analyzed the effect of adjuvant chemotherapy in HR-positive patients; however, the differential efficacy of adjuvant chemotherapy or not between HR-negative ILC and IDC has not been investigated. In agreement with previous results, among patients who received chemotherapy, we found that the ILC group had a worse prognosis compared to the IDC group in HR-negative patients (HR 1.469; 95% CI 1.093–1.973; p=0.01), whereas the ILC and IDC groups had similar OS in HR-positive patients (HR 0.992; 95% CI 0.905–1.088; p=0.871).

Some limitations of our study should to be considered. One limitation of this study is that we were unable to distinguish between ‘‘pure’’ ILC and ‘‘mixed’’ type ILC among the different geographic regions of SEER. Each histological subtype has different outcomes in ILC, for example, pleomorphic ILC with aggressive clinical features has a worse prognosis than classical ILC (7, 30). Second, our study is a retrospective cohort study performed using the SEER database, so there is selection bias and missing data.

In conclusion, our research demonstrated that there was no significant difference in overall survival between ILC and IDC patients after matching for several known covariates. In the subgroup analysis stratified by negative hormone receptor and positive lymph node status, we found that the prognosis of ILC was significantly worse compared to IDC. Among patients who received chemotherapy, the ILC group had worse prognosis compared to the IDC group in HR-negative patients, whereas the ILC and IDC groups exhibited similar OS in HR-positive patients. These results may indicate that we should enhance treatment among these special subgroups to prolong survival.

## Data Availability Statement

Publicly available datasets were analyzed in this study. This data can be found here: Surveillance, Epidemiology, and End Results (SEER) database (https://seer.cancer.gov/).

## Ethics Statement

The requirement for informed consent was waived because personal information of patients was not involved.

## Author Controbutions

CY, CL, and YZ contributed to the idea and design. JZ, FJ, WP, LZ, HG, MY, and JL contributed to the data acquisition and analysis. CY, CL, and KW contributed to the manuscript writing and revision. All authors contributed to the article and approved the submitted version.

## Funding

Guangdong Basic and Applied Basic Research Foundation (grant number 2020A1515010346), Guangdong Medical Science and Technology Research Fund (grant number A2019494, A2019252), and The Doctor Launch Fund of Guangdong Provincial People’s hospital (grant number 2018bq04).

## Conflict of Interest

The authors declare that the research was conducted in the absence of any commercial or financial relationships that could be construed as a potential conflict of interest.
